# Optimal Isolation Method of Small Extracellular Vesicles from Rat Plasma

**DOI:** 10.3390/ijms20194780

**Published:** 2019-09-26

**Authors:** Kosuke Otani, Yusei Fujioka, Muneyoshi Okada, Hideyuki Yamawaki

**Affiliations:** Laboratory of Veterinary Pharmacology, School of Veterinary Medicine, Kitasato University, Towada, Aomori 034-8628, Japan; dv16002@st.kitasato-u.ac.jp (K.O.); vm14116a@st.kitasato-u.ac.jp (Y.F.); mokada@vmas.kitasato-u.ac.jp (M.O.)

**Keywords:** extracellular vesicles, plasma, rat, polyethylene-glycol, anticoagulant

## Abstract

Small extracellular vesicles (sEVs) mediate cell–to–cell communication. We recently reported that circulating sEVs regulate systolic blood pressure in an animal model of human systemic hypertension. However, the underlying mechanisms still remain to be elucidated. As the first step for detailed analyses, we sought to increase the yield and purity of sEVs isolated from rat plasma. We compared the concentration and size distribution of sEVs as well as protein expression of the sEV marker and contaminants among plasma sEVs isolated by the ultracentrifugation (UC) method, the precipitation with polyethylene-glycol and ultracentrifugation (PEG-UC) method, or the precipitation with polyethylene-glycol (PEG) method. Effects of anticoagulants were also examined. The total concentration of plasma sEVs isolated by the PEG or PEG-UC method was much higher than that of the UC method. In the plasma sEVs isolated by the PEG-UC method, contaminating proteins were lower, while the protein expression of certain sEV markers was higher than that of the PEG method. There was no significant difference in total concentration or protein expression of sEV markers in sEVs isolated from rat plasma treated with three different anticoagulants (heparin, ethylenediaminetetraacetic acid, or acid citrate dextrose buffer) by the PEG-UC method. We, for the first time, determined that the PEG-UC method was optimal for sEV isolation from rat plasma.

## 1. Introduction

Cells release lipid-bilayer-capsuled particles containing various molecules, such as proteins, DNA, mRNA, small RNA, and others, into extracellular fluid [1,2]. These “molecular cargoes”, namely extracellular vesicles (EVs), were typically classified as small (sEVs or exosome, with a diameter of approximately 50–150 nm) or large EVs (or microvesicle, with a diameter of approximately 50 nm–1 μm) [3]. Released sEVs affect cellular function via signal transduction by binding to cell surface receptors and delivery of the baggage by phagocytosis, pinocytosis, or membrane fusion. Therefore, sEVs are recognized as the active particles mediating cell–to–cell communication [4]. Recent studies suggest that sEVs play a key role in various disease states, including cancer [5], disorders in the central nervous system [6], and cardiovascular disease [7]. Recently, we also reported that plasma sEVs in a spontaneously hypertensive rat (SHR), an animal model of human systemic hypertension, regulate systolic blood pressure [8]. However, the mechanisms of action of sEVs in the pathogenesis of hypertension in an SHR still remain to be elucidated. It is, thus, necessary to increase the yield and purity of sEVs isolated from plasma for the detailed examination.

sEVs are often isolated by ultracentrifugation, density gradient centrifugation, size exclusion chromatography, or affinity purification with specific antibodies to sEV marker proteins from body fluid and cell-culture media [9,10]. Currently, novel methods for sEV isolation, such as size-based exclusion by using a modular platform [11], acoustic purification by using a microfluid device [12,13], and others [14], have been developed, because by using conventional methods it is not possible to completely achieve enough quality. Today, the effectiveness of the precipitation method by using polyethylene-glycol for sEV isolation derived from human plasma and cell-culture media is also reported [15]. However, it remains to be determined whether it is useful for sEV isolation from rat plasma. Then, we sought to explore it in this study.

Plasma can be separated from the blood sample mixed with an anticoagulant. Common anticoagulants include heparin, ethylenediaminetetraacetic acid (EDTA), acid citrate dextrose buffer (ACD), and others. It was demonstrated that EDTA or ACD inhibits secretion of large EVs from human platelet during isolation procedures [16,17]. It was also indicated that anticoagulants affect sEV population in human plasma [18]. However, little is known about the effects of anticoagulants on plasma sEVs, especially from rats. Therefore, we also examined whether anticoagulants may affect the yield and purity of sEVs derived from rat plasma.

## 2. Results

### 2.1. Concentration and Size Distribution of Plasma sEVs Isolated by Three Methods

**Figure 1:**
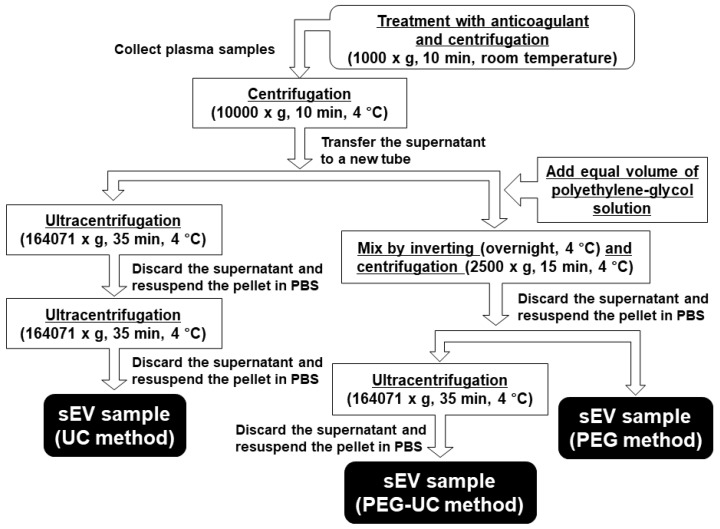
Scheme of isolation procedures for small extracellular vesicles (sEVs) derived from rat plasma. Blood samples were obtained from male Wistar rats (7–9-week-old) via an inferior vena cava under anesthetization with urethane (1.5 g/kg, i. p.). Ethylenediaminetetraacetic acid (EDTA, 1 mg/mL)-anticoagulated blood samples were separated into plasma by centrifugation (1000× *g*, 10 min, room temperature). The plasma sample derived from one Wistar rat was divided equally into three groups including the ultracentrifugation (UC) method, precipitation with polyethylene-glycol and ultracentrifugation (PEG-UC) method, or precipitation with polyethylene-glycol (PEG) method after depletion of large EVs by centrifugation (10,000× *g*, 10 min, 4 °C). PBS: phosphate-buffered saline.

We first examined the differences in concentration and size distribution of plasma sEVs isolated by three different methods; the ultracentrifugation (UC) method, precipitation with polyethylene-glycol and ultracentrifugation (PEG-UC) method, or precipitation with polyethylene-glycol (PEG) method, as described in Figure 1.

**Figure 2:**
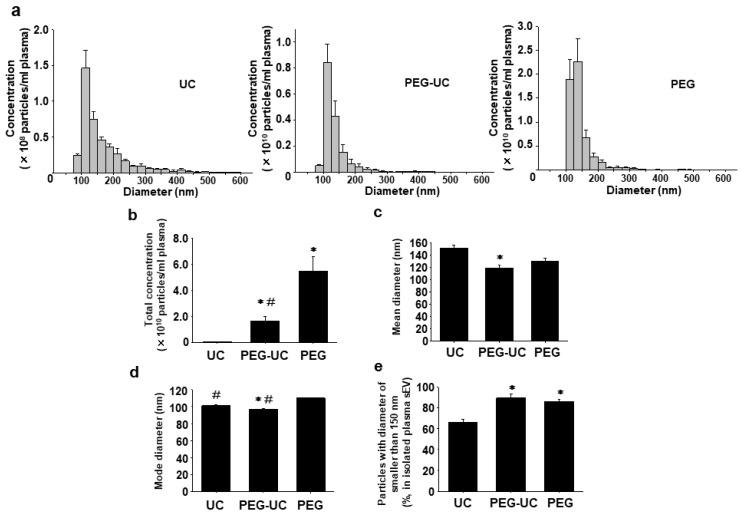
Concentration and size distribution of plasma sEVs isolated by three (UC, PEG-UC, or PEG) methods. sEVs were isolated from the EDTA (1 mg/mL)-anticoagulated plasma in male Wistar rats (7–9-week-old) by the three methods as described in Figure 1. Concentration and size distribution of sEVs were measured by a tunable resistive pulse sensing analyses (TRPS) method using a qNANO instrument. (**a**) Concentration and size distribution of plasma sEVs were shown. The concentration was normalized to the starting volume of plasma (particles/mL). (**b**) Total concentration of plasma sEVs was shown (particles/mL). (**c**,**d**) Mean and mode (most frequent) diameters of plasma sEVs were shown. (**e**) The percentage of particles with a diameter of smaller than 150 nm in total particles of plasma sEVs was shown. Results were expressed as means ± standard error of the mean (SEM) in bar graphs (*n* = 4). * *p* < 0.05 vs. UC, # *p* < 0.05 vs. PEG.

In each method, particle size was distributed within the expected range [19] with a diameter of 100–150 nm (Figure 2a, *n* = 4).

Total concentration of plasma sEVs was highest in the PEG method, while it was lowest in the UC method (Figure 2b, *n* = 4, 5.43 ± 1.12 × 10^10^ particles/mL in PEG > 1.63 ± 0.34 × 10^10^ particles/mL in PEG-UC >> 4.17 ± 1.12 × 10^8^ particles/mL in UC). In the PEG-UC method, the mean diameter of plasma sEVs was significantly smaller than that in the UC method (Figure 2c, *n* = 4, 118.8 ± 4.8 nm in PEG-UC, *p* < 0.05 vs. 150.8 ± 5.5 nm in UC). A mode diameter, the most frequent particle size of plasma sEVs in the UC or PEG-UC method was significantly smaller than that in the PEG method (Figure 2d, *n* = 4, 101.0 ± 0.9 nm in UC or 96.5 ± 1.6 nm in PEG-UC, *p* < 0.05 vs. 110.0 ± 0.8 nm in PEG). Percentage of particles with a diameter of smaller than 150 nm in the PEG-UC or PEG method was significantly higher than that in the UC method (Figure 2e, *n* = 4, 89.3 ± 3.6% in PEG-UC or 86.0 ± 2.3% in PEG, *p* < 0.05 vs. 65.8 ± 3.0% in UC).

### 2.2. Total Protein Concentration and Expression of Marker Proteins for sEVs (CD81, CD9, and CD63), Large EVs (α-Actinin-4), and Plasma (Albumin) in sEVs Isolated by the Different Isolation Methods

**Figure 3:**
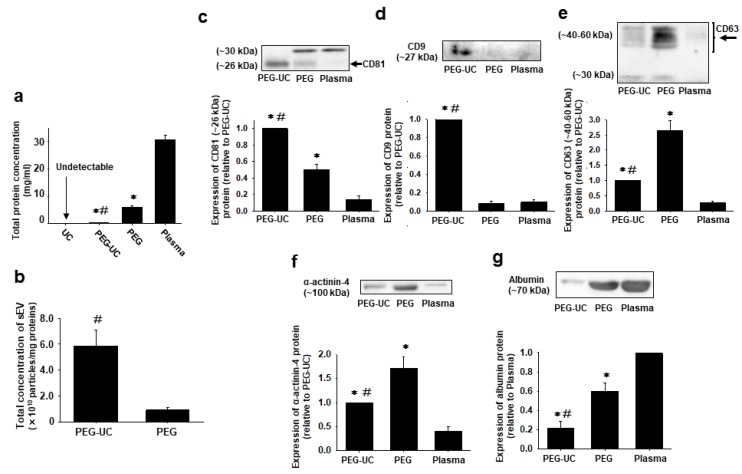
Total protein concentration and expression of marker proteins for sEVs (CD81, CD9, and CD63), large EVs (α-actinin-4), or plasma (albumin) in sEVs isolated by the different methods as described in Figure 1. Total protein was extracted from plasma sEVs of male Wistar rats (7–9-week-old) using radio-immunoprecipitation assay (RIPA) buffer. (**a**) The concentration of proteins was measured by a bicinchoninic acid (BCA) assay and normalized to the starting volume of plasma (mg/mL). (**b**) The total concentration of plasma sEVs normalized to each protein concentration was shown (particles/mg). (**c**–**g**) Expression of marker proteins in plasma sEVs was determined by Western blotting using an antibody to CD81, CD9, CD63, α-actinin-4, or albumin. The arrows indicated the bands for CD81 (c) and CD63 (e) that we measured for quantitative analyses. Data were shown as fold increase relative to the expression level in sEVs isolated by the PEG-UC method (c: CD81, d: CD9, e: CD63, f: α-actinin-4) or plasma (g: albumin). Results were expressed as means ± SEM in bar graphs (*n* = 4). Uncropped images are shown in Supplementary Figure S2. * *p* < 0.05 vs. plasma, # *p* < 0.05 vs. PEG.

We next determined the protein expression in isolated sEVs. The total protein concentration of plasma sEVs in the PEG-UC method was significantly lower than that in the PEG method (Figure 3a, *n* = 4, 0.28 ± 0.04 mg/mL in PEG-UC, *p* < 0.01 vs. 6.02 ± 0.51 mg/mL in PEG), while that in the UC method was under the detectable range of a bicinchoninic acid (BCA) assay.

In the PEG-UC method, the particle concentration of the plasma sEVs normalized to protein concentration was significantly higher than that in the PEG method (Figure 3b, *n* = 4, 5.88 ± 2.42 × 10^10^ particles/mg in PEG-UC, *p* < 0.05 vs. 0.91 ± 0.37 × 10^10^ particles/mg in PEG). The protein expression of the sEV marker, CD81 [19] (~26 kDa, an expected size), in plasma sEVs isolated by the PEG-UC method was significantly higher than that isolated by the PEG method (Figure 3c, *n* = 4, *p* < 0.05). Of note, we detected another band (~30 kDa), which was higher than the expected size for CD81. The protein expression of the sEV marker, CD9 [19], in plasma sEVs isolated by the PEG-UC method was significantly higher than that isolated by the PEG method (Figure 3d, *n* = 4, *p* < 0.05). In contrast, the protein expression of the sEV marker, CD63 [19] (~40–60 kDa, an expected size) in plasma sEVs isolated by the PEG-UC method was significantly lower than that isolated by the PEG method (Figure 3e, *n* = 4, *p* < 0.05). We also detected a band (~30 kDa), which was lower than the expected size for CD63. The protein expression of a large EV marker, α-actinin-4 [20] and plasma albumin in sEVs isolated by the PEG-UC method was significantly lower than that isolated by the PEG method (α-actinin-4: Figure 3f, *n* = 4, *p* < 0.05; albumin: Figure 3g, *n* = 4, *p* < 0.05).

### 2.3. Effects of Anticoagulants on Concentration and Size Distribution of Plasma sEVs

**Figure 4:**
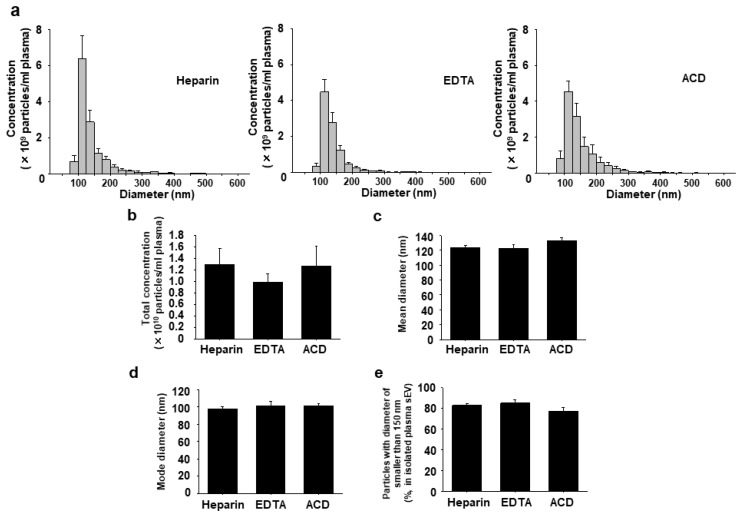
Effects of anticoagulants on concentration and size distribution of plasma sEVs. sEVs were isolated from heparin (1 U/mL)-, EDTA (1 mg/mL)-, or acid citrate dextrose buffer (ACD, 13%)-anticoagulated plasma of Wistar rats (5–10-week-old) by the PEG-UC method. Concentration and size distribution of sEVs were measured by a TRPS method using a qNANO instrument. (**a**) Concentration and size distribution of plasma sEVs were shown (particles/mL). The concentration was normalized to the starting volume of plasma. (**b**) The total concentration of plasma sEVs was shown (particles/mL). (**c**, **d**) Mean and mode (most frequent) diameters of plasma sEVs were shown. (**e**) The percentage of particles with a diameter of smaller than 150 nm in total particles of plasma sEVs was shown. Results were expressed as means ± SEM in bar graphs (*n* = 4).

Next, we isolated sEVs from rat plasma treated with three different anticoagulants, heparin, EDTA, or ACD, by the PEG-UC method, and compared concentration and size distribution. Plasma sEVs were distributed within the expected range with a diameter of 100–150 nm, irrespective of the anticoagulants (Figure 4a, *n* = 4).

There was no significant difference in total concentration, mean diameter, mode diameter, or percentage of particles with a diameter of smaller than 150 nm in plasma sEVs among the three different anticoagulants (Figure 4b–e, *n* = 4).

### 2.4. Effects of Anticoagulants on Total Protein Concentration and Expression of Marker Proteins for sEVs, Large EVs, and Plasma in sEVs

**Figure 5:**
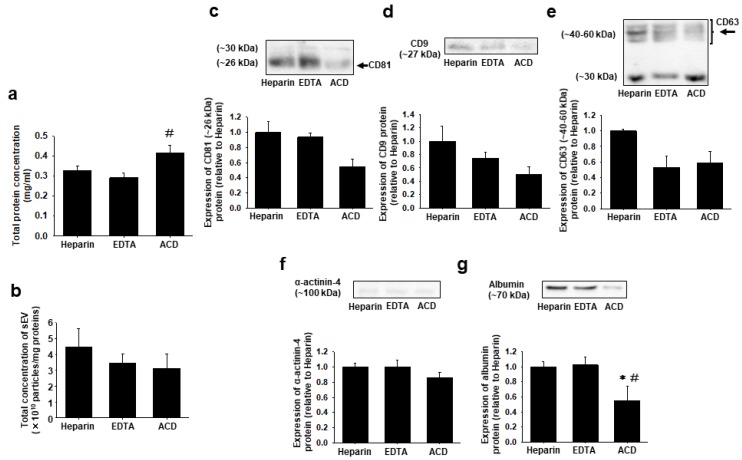
Effects of anticoagulants on total protein concentration and expression of marker proteins for sEVs, large EV, or plasma in sEVs. Total protein was extracted from sEVs isolated from heparin (1 U/mL)-, EDTA (1 mg/mL)-, or ACD (13%)-anticoagulated plasma in Wistar rats (5–10-week-old) by the PEG-UC method using RIPA buffer. (**a**) The concentration of proteins was measured by a BCA assay and normalized to the starting volume of plasma (mg/mL). (**b**) The total concentration of plasma sEVs normalized to each protein concentration was shown (particles/mg). (**c**–**g**) Expression of marker proteins was determined by Western blotting using antibody to CD81, CD9, CD63, α-actinin-4, or albumin. The arrows indicated the bands for CD81 (c) and CD63 (e) that we measured for quantitative analyses. Data were shown as fold increase relative to the expression level in sEVs derived from the heparin-anticoagulated plasma. Results were expressed as means ± SEM in bar graphs (*n* = 4). Uncropped images are shown in Supplementary Figure S3. * *p* < 0.05 vs. heparin, # *p* < 0.05 vs. EDTA.

The total protein concentration of sEVs from the ACD-treated plasma was significantly higher than that from the EDTA-treated plasma (Figure 5a, *n* = 4, 0.42 ± 0.03 mg/mL in ACD, *p* < 0.05 vs. 0.29 ± 0.02 mg/mL in EDTA).

There was no significant difference in particle concentration normalized to protein concentration in plasma sEVs among the three different anticoagulants (Figure 5b, *n* = 4). There was no significant difference in expression of sEV marker proteins (CD81: ~26 kDa; CD9; CD63: ~40–60 kDa) in plasma sEVs among the three different anticoagulants (Figure 5c–e, *n* = 4). The expression of α-actinin-4 protein was scarcely detected in each plasma sEV (Figure 5f, *n* = 4). The expression of albumin protein in sEVs from the ACD-treated plasma was significantly lower than that from the heparin- or EDTA-treated plasma (Figure 5g, *n* = 4, 0.55 ± 0.19-fold relative to heparin, *p* < 0.05). We also examined the effects on platelet activation and found that the protein expression of the platelet marker was negative in the three different anticoagulants-treated plasma sEV samples (Supplementary Figure S1).

## 3. Discussion

In the present study, we examined firstly whether the PEG-based precipitation was effective for isolation of sEVs derived from rat plasma and secondly the effects of anticoagulants on the yield and purity of plasma sEVs. Then, we for the first time, demonstrated that sEVs isolated from rat plasma by the PEG-based method in combination with ultracentrifugation could be optimal in terms of high yield and purity. We also showed that the effects of anticoagulants are minimal. We confirmed in the electron microscopic observation for sEVs isolated by the PEG-UC method from the heparin-treated rat plasma that the sEVs showed a typical capped structure with an appropriate size (~100 nm) (Supplementary Figure S4).

Consistent with a previous report [15], the total concentration of plasma sEVs was the highest in the PEG method, while it was the lowest in the UC method (Figure 2b, PEG > PEG-UC >> UC). It is thus suggested that the yield of sEVs is certainly reduced through an ultracentrifugation step [21]. Moreover, the aggregation of sEVs isolated by ultracentrifugation was observed [22]. It is thus speculated that this is a possible reason why sEVs in the UC method represented a larger size distribution in the present study (Figure 2a,c,e). On the other hand, the contaminations of large EV marker protein (α-actinin-4) and plasma albumin in sEVs isolated by the PEG-UC method were much lower than that by the PEG method (Figure 3f,g). These results indicate that a washing procedure by ultracentrifugation is effective to eliminate contaminants and also to enhance the purity of isolated sEVs as previously reported [15,23].

It is demonstrated that sEVs obtained by a polymer-based commercial precipitation kit contained abundant contaminants, including aggregated proteins [24,25]. Basically, a principal of these kits is similar to the PEG method in the present study where the aqueous solubility of sEVs is reduced [15]. A previous report demonstrated that it is possible to remove contaminants from sEVs isolated by the commercial kits through washing by an additional ultracentrifugation step [23]. Then, it is suggested in the isolation of sEVs from rat plasma that the PEG-UC could be a cost-effective alternative method to the commercial kits.

There was no significant difference in the total concentration of plasma sEVs among the three different anticoagulants (Figure 4b). Contrastingly, a previous report demonstrated that the concentration of sEVs from the EDTA-treated plasma was higher than that from other anticoagulants-treated plasmas [18]. In the study, however, the concentration and size of sEVs were measured by a nanoparticle tracking analysis after freeze and thaw, which may potentially affect the concentration and size distribution of sEVs [26,27]. We speculate that this is one possible explanation for the discrepancy. There was no significant difference in the percentage of particles with a diameter of larger than 150 nm in plasma sEVs among the three different anticoagulants (Figure 4e). Contrastingly, it was reported that the amount of large EVs in the ACD-treated plasma was lower than that in the heparin-treated plasma [16,17,18]. In the study, large EVs in the plasma were directly detected by a flow cytometry, while sEVs were not detected. This is one possible explanation for the discrepancy, and we believe that our results represent a more accurate population of plasma sEVs.

In sEVs from the ACD-treated plasma, the total protein concentration was significantly higher than that from the EDTA-treated plasma, while plasma albumin expression was significantly lower compared with other plasma preparations (Figure 5a,g). It is assumed that ACD may cause a precipitation of other plasma proteins than albumin and/or a sticking of these proteins on the sEV surface during the isolation procedure of the PEG-UC method. Little is known about the effects of anticoagulants on co-precipitated proteins with plasma sEVs. A recent report, however, demonstrated that an addition of albumin could prevent sEVs from sticking to other materials [28], supporting the present results.

CD81, CD9, and CD63 proteins are widely recognized as sEV marker proteins [19]. The expression of CD81 and CD9 proteins in sEVs isolated by the PEG-UC method was significantly higher than that by the PEG method, while the expression of the CD63 protein in sEVs isolated by the PEG-UC method was significantly lower than that by the PEG method (Figure 3c–e). It should be noted that we can not exclude the possibility that the CD9 antibody used in this study had low cross-reactivity to rat CD9. Nonetheless, it was previously reported that sEV marker proteins are not expressed in parallel, while they show different patterns depending on donor cells, disease states, and isolation procedures [29,30,31], supporting our results. In addition, the loading of CD81, CD9, and CD63 onto sEV membranes via different pathways was suggested [32,33]. Therefore, comprehensive identification of sEVs by analyzing the expression of marker proteins, size distribution, morphology, and function would be necessary.

In conclusion, we, for the first time, demonstrated that the isolation method by ultracentrifugation following precipitation with polyethylene-glycol enables to achieve high yield and purity in sEVs from rat plasma. In addition, the effects of anticoagulants are minimal. Our findings could contribute to better understanding mechanisms underlying various diseases, including hypertension, diabetes, obesity, and others, mediated by circulating sEVs, especially in rat models.

## 4. Materials and Methods

### 4.1. Animals and Plasma Sample Collection

Animal care and procedures were performed in conformity with the institutional guideline of the School of Veterinary Medicine, Kitasato University. The animal study was approved by the ethical committee of the School of Veterinary Medicine, Kitasato University. Male Wistar rats (5–10-week-old) (CLEA Japan, Tokyo, Japan) were maintained in a constant temperature and humidity room (22 ± 2 °C, 50–60%, 12 h for lighting). They can freely take food (CE2, CLEA) and water. After the rats were anesthetized deeply with urethane (1.5 g/kg, i.p., Sigma-Aldrich, St. Louis, MO, USA), blood was drawn via an inferior vena cava using a 5 mL syringe (JMS, Hiroshima, Japan) and a 20 G needle (JMS) coated with an anticoagulant, heparin (1000 U/mL, AY Pharmaceuticals, Tokyo, Japan), EDTA (100 mg/mL, Sigma-Aldrich), or ACD. The ACD consisted of 0.23 mg/mL tri-sodium citrate dihydrate (KANTO CHEMICAL, Tokyo, Japan), 0.14 mg/mL citric acid (FUJIFILM Wako Pure Chemical, Osaka, Japan), and 0.2 mg/mL glucose (KANTO CHEMICAL) in ultra-pure ddH_2_O. The collected blood samples were treated with anticoagulant (heparin at a final concentration of 1 U/mL, EDTA at a final concentration of 1 mg/mL, or ACD at a final concentration of 13%). They were gently mixed and centrifuged (1000× *g*, 10 min, room temperature) by using a microcentrifuge (model 3740, Kubota Corp., Tokyo, Japan) to separate plasma. Plasma samples were frozen in liquid nitrogen and stored at −80 °C until further experiments.

### 4.2. Isolation Procedures for sEVs Derived from Rat Plasma

The overview of isolation procedures was described in a graphical scheme (Figure 1). Plasma samples were thawed at 37 °C and centrifuged (10,000× *g*, 10 min, 4 °C) to eliminate large EVs by using the microcentrifuge. The supernatant was divided equally into three groups, including the UC method, PEG-UC method, or PEG method. The UC method was performed as previously described [8]. Briefly, the supernatant was ultracentrifuged (164,071× *g*, 35 min, 4 °C) by using an Optima XL-80K ultracentrifuge with a swing rotor SW 55 Ti (Beckman Coulter Inc., Miami, FL, USA). The pellet was resuspended in phosphate-buffered saline (PBS, Sigma-Aldrich) and ultracentrifuged again. Then, the pellet was resuspended again in PBS, which was used for the following analysis. The PEG-UC and PEG methods were performed as described in the previous report [15]. In the PEG-UC method, the large EV-depleted supernatant was mixed with an equal volume of polyethylene-glycol solution consisting of 16% polyethylene-glycol (MW = 6000, FUJIFILM Wako Pure Chemical) and 1 M NaCl (Nacalai Tesque, Kyoto, Japan) by inverting (overnight, 4 °C). The mixture was then centrifuged (2500× *g*, 15 min, 4 °C), and the supernatant was discarded. The pellet was resuspended in PBS by vigorous vortex (30 min, room temperature) and ultracentrifuged (164,071× *g*, 35 min, 4 °C). The pellet was resuspended again in PBS, which was used for the following examination. In the PEG method, the large EV-depleted supernatant was mixed with an equal volume of polyethylene-glycol solution by inverting (overnight, 4 °C) and centrifuged (2500× *g*, 15 min, 4 °C). The pellet was resuspended in PBS by vigorous vortex (30 min, room temperature), which was used for the following examination.

### 4.3. Concentration and Size Distribution of Plasma sEVs

The concentration and size distribution of isolated sEVs were measured by a tunable resistive pulse sensing (TRPS) method using a qNANO instrument with an NP100 or NP150 nanopore at a 46.0–47.0 mm stretch (IZON Science, Christchurch, New Zealand) according to the manufacturer’s instruction. The nanopores were coated with a TRPS Reagent Kit (IZON Science) for the prevention of sEV adhesion. Raw data were standardized by carboxylated-polystyrene particles with a diameter of 110 nm, of which the concentration was known.

### 4.4. Protein Expression in Plasma sEVs

Protein lysates were extracted from plasma sEVs using a radio-immunoprecipitation assay buffer containing 20 mM Tris-HCl (pH7.5), 150 mM NaCl, 10 mM MgCl_2_, 1% Triton-X, 0.5% sodium deoxycholate, 0.1% sodium dodecyl sulfate, 1% protease inhibitor mixture, and 1% phosphatase inhibitor mixture (Nacalai Tesque) on ice. Protein concentration was determined by a BCA assay (Pierce, Rockford, IL, USA). Western blotting was performed as previously described [34]. After equal amounts of proteins (3.5–10 µg) were separated by SDS-PAGE (7.5–14%, 80–120 V, 1.5 h), they were transferred (400 mA, 1.5 h) to a nitrocellulose membrane (Pall Corporation, Ann Arbor, MI, USA). For confirming the equal loading of protein, the membranes were stained with 0.1% Ponceau-S/5% acetic acid (5 min, room temperature) and washed with 1% acetic acid (three times, 5 min, room temperature). The Ponceau-S stained membranes were scanned in visible light by using the ATTO light capture system (ATTO, Tokyo, Japan). The total density of all the visible bands in each lane was measured as the amount of total protein using CS analyzer 3.0 software (ATTO). After being blocked with 0.5% skim milk, the membranes were incubated with a primary antibody [1:500 dilution in Tris-buffered saline with 0.1% Tween 20 (TBS-T)] (overnight, 4 °C). They were detected by using HRP-conjugated secondary antibody (1:10,000 dilution in TBS-T, 1 h, room temperature) and the EZ-ECL reagent (Biological Industries, Kibbutz Beit Hesmek, Israel). The results were analyzed using CS analyzer 3.0 software. The antibody sources for Western blotting were as follows: rabbit antibodies to CD81 (EXOAB-CD81A-1) and CD63 (EXOAB-CD63A-1, System Biosciences, Palo Alto, CA, USA); mouse antibody to CD9 (#60232, Proteintech, Rosemont, IL, USA); mouse antibodies to α-actinin-4 (sc-393495) and albumin (sc-270165, Santa Cruz Biotech, Santa Cruz, CA, USA); HRP-liked secondary goat antibody to rabbit IgG (EXOAB-HRP, System Biosciences), and sheep antibody to mouse IgG (NA931, GE Healthcare, Chicago, IL, USA).

### 4.5. Statistical Analysis

Data were shown as means ± standard error of the mean. Statistical evaluations were performed using a one-way nonparametric test by the Kruskal-Wallis method followed by Mann–Whitney’s U test for multiple comparisons. The comparison of the two groups was performed by Mann–Whitney’s U test. Values of *p* < 0.05 were considered statistically significant.

## Supplementary Materials

Supplementary materials can be found at https://www.mdpi.com/1422-0067/20/19/4780/s1.

## Abbreviations

EV	Extracellular vesicle
sEV	Small EV
SHR	Spontaneously hypertensive rat
EDTA	Ethylenediaminetetraacetic acid
ACD	Acid citrate dextrose buffer
UC	Ultracentrifugation
PEG-UC	Precipitation with polyethylene-glycol and ultracentrifugation
PEG	Precipitation with polyethylene-glycol
TRPS	Tunable resistive pulse sensing
SEM	Standard error of the mean
BCA	Bicinchoninic acid
RIPA	Radio-immunoprecipitation assay
PBS	Phosphate-buffered saline
TBS-T	Tris-buffered saline with 0.1% Tween 20
