# Supplementary material for: Optimal Isolation Method of Small Extracellular Vesicles from Rat Plasma
Source: Int J Mol Sci. 2019 Sep 26;20(19):4780. doi: 10.3390/ijms20194780 (PMC6801590; doi:10.3390/ijms20194780)
Supplement: Supplementary file 1 [file ijms-20-04780-s001.pdf]

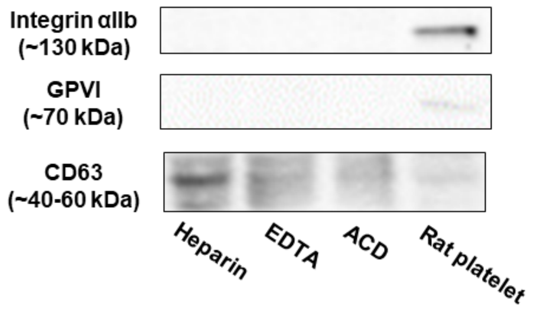

Supplementary Figure 1. Expression of marker proteins for platelet (integrin αIIb and GPVI) in small extracellular vesicles (sEV) from anticoagulated plasma. Total protein was extracted from sEV isolated from heparin (1 U/ml)-, EDTA (1 mg/ml)-, or ACD (13%)-anticoagulated plasma of Wistar rats (5-10-week-old) by a precipitation with polyethylene-glycol and ultracentrifugation (PEG-UC) method. The expression of protein was determined by Western blotting using an antibody to integrin αIIb and GPVI (a platelet marker) or CD63 (an sEV marker). Protein extract of rat platelet was loaded as a positive control for expression of platelet marker proteins. Representative images were shown (n = 4). CD63 blot in this Figure was from the same origin as shown in Fig. 5e.

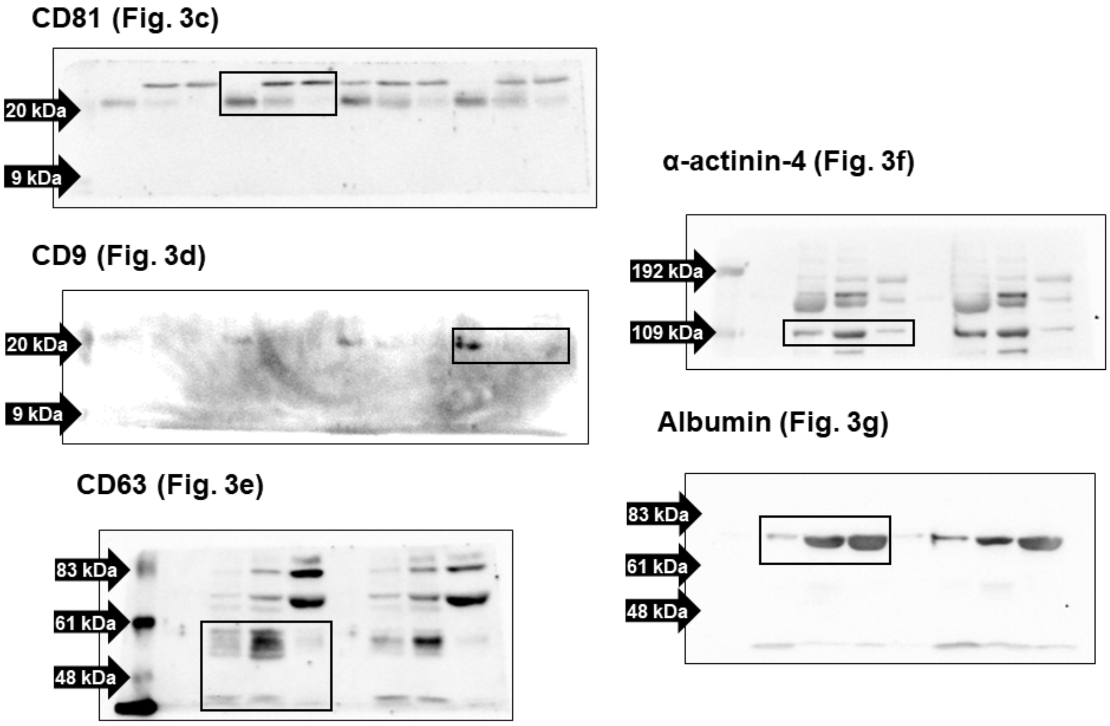

<u>Supplementary Figure 2.</u> Uncropped blot images by Western blotting in Figure 3c-3g. Black arrows indicate the bands of molecular weight marker, and black-edged frames show the cropped areas represented in Figure 3c-3g.

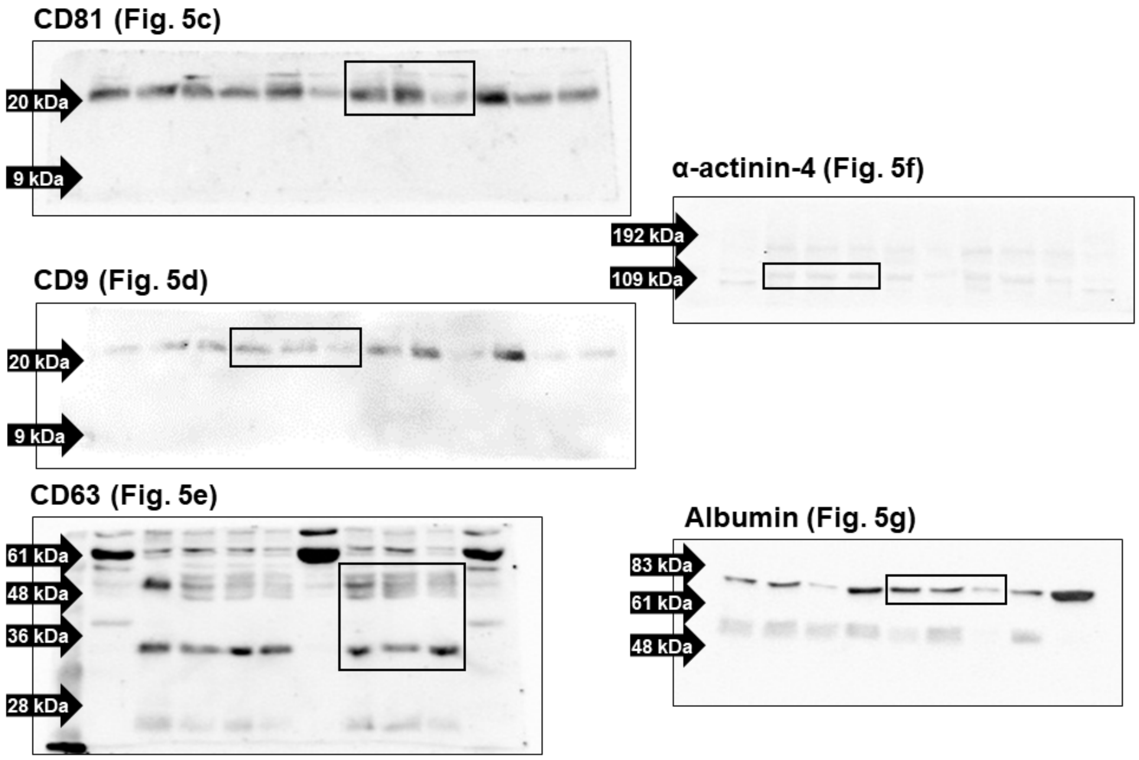

<u>**Supplementary Figure 3.**</u> Uncropped blot images by Western blotting in Figure 5c-5g. Black arrows indicate the bands of molecular weight marker, and black-edged frames show the cropped areas represented in Figure 5c-5g.

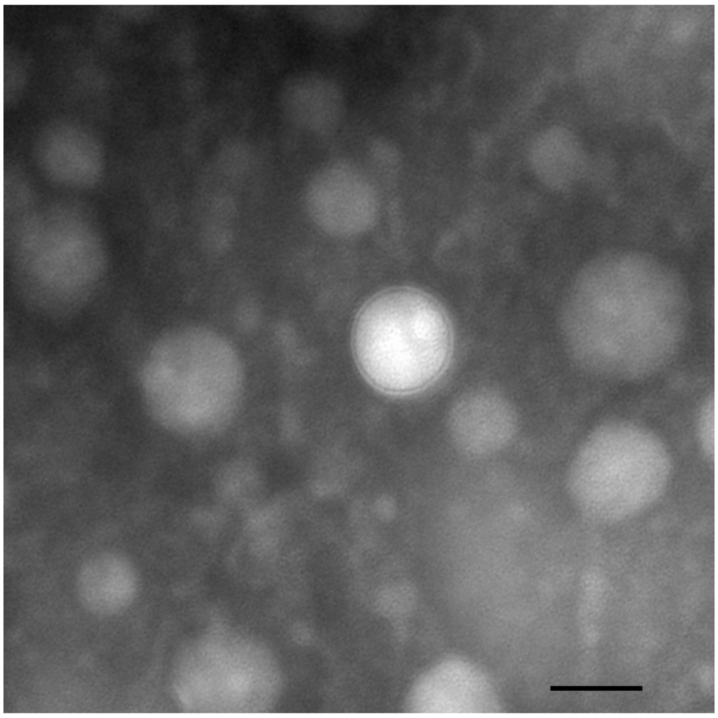

<u>Supplementary Figure 4.</u> Microscopic evaluation of sEV by using a transmission electronic microscope (TEM). sEV isolated from heparin (1 U/ml)-anticoagulated plasma of Wistar rats (6-week-old) by a PEG-UC method were fixed with 2% paraformaldehyde. The fixed sEV were stained with phosphotungstic acid on a carbon-coated copper grid and observed by using TEM (80 MeV, × 15000). Scale bar: 100 nm.
